# Interaction between functional capability and sleep quality at midterm after total knee arthroplasty: a Japanese retrospective cohort study

**DOI:** 10.1038/s41598-023-45603-4

**Published:** 2023-10-26

**Authors:** Satoshi Hamai, Satoru Harada, Hidetoshi Tsushima, Ryutaro Kozuma, Satoshi Yamate, Shinya Kawahara, Yukio Akasaki, Tetsunari Harada, Yasuhiko Kokubu, Toshiki Konishi, Yasuharu Nakashima

**Affiliations:** 1 Department of Medical-Engineering Collaboration for Healthy Longevity, Faculty of Medical Sciences, Kyushu University, Fukuoka, Japan; 2 Department of Orthopedic Surgery, Faculty of Medical Sciences, Kyushu University, Fukuoka, Japan

**Keywords:** Diseases, Medical research, Risk factors

## Abstract

No report has clarified the frequency and interacting factors affecting sleep disturbance among Asian patients at midterm after total knee arthroplasty (TKA). This study aimed to evaluate the frequency of sleep disturbance at midterm after TKA in a Japanese cohort and to identify intervening factors for sleep. We hypothesized that residual knee pain and decreased functional capability negatively interact with sleep quality after TKA. A total of 209 Japanese participants (average age: 77.1 ± 8.3 years; postoperative follow-up period: 4.5 ± 1.9 years) who underwent primary TKA for knee osteoarthritis were included in this study. Sleep quality, satisfaction, pain, functional capability, joint awareness, and mental condition were evaluated using the Pittsburgh Sleep Quality Index (PSQI), Knee Society Score (KSS) 2011, Forgotten Joint Score (FJS)-12, and 12-Item Short-Form Health Survey (SF-12) Mental Component Summary (MCS). Multivariable analysis was performed to determine the influencing factors on PSQI. The scores for the PSQI, satisfaction with pain level while lying in bed, pain during level walking, functional activity category in the KSS 2011, awareness of the artificial joint in bed at night in the FJS-12, and SF-12 MCS were 6.7 ± 3.0, 5.8 ± 1.8, 1.6 ± 2.3, 62 ± 22, 1.5 ± 1.4, and 56 ± 9.3 on average, respectively. Sleep disturbance (PSQI ≥ 5.5) occurred in 54% of the Japanese participants. Multivariable analysis revealed that high functional capability was a significant factor associated with sleep quality improvement (p < 0.05). Decreased functional capability, not residual knee pain, negatively interacted with sleep quality. The sleep disturbance rate was high during the middle postoperative period after TKA in the Japanese cohort.

## Introduction

Osteoarthritis (OA) is a common chronic condition that presents with pain and dysfunction^1^. Symptomatic knee OA occurs in 10% and 13% of men and women aged ≥ 60 years, respectively^2^, and these rates are likely to increase as a result of the obesity epidemic and population aging worldwide. In Japan, more than ten million people have been estimated to suffer from knee OA^3^.

Sleep disturbance is common among adults, particularly in the Japanese cohort^4^, and seriously affects the quality of life (QOL), which is worsened by OA^5^. Sleep disturbances in Japan can be attributed to a combination of cultural, societal, and lifestyle factors (e.g., long working hours, noise and light pollution in urban areas, chronic stress due to competitive education system and job market, and lifestyle habits from a young age)^6,7^. In 2019, the Organization for Economic Co-operation and Development reported that, among 33 countries, Japan had the shortest sleep duration (7 h, 22 min), which was 1 h shorter than the average time. Furthermore, approximately 30–80% of patients with knee OA have been reported to suffer from insomnia^8^. Knee OA-related insomnia could be linked to both greater pain and decreased physical function. Central pain modulatory processes are increasingly recognized as a possible underlying mechanism linking disturbed sleep and pain^9^.

Total knee arthroplasty (TKA) is considered an effective treatment for patients with knee OA, providing pain relief, improving the gait, and restoring the activities of daily living^10–14^. Several previous prospective studies from Western countries have investigated sleep disturbance before and after TKA^15–23^. In particular, one previous study revealed that insomnia occurred in approximately 50% of patients in the short term after TKA and was associated with knee pain^19^. Nonetheless, only few studies have examined the frequency of sleep disturbance in Asian cohorts after TKA or investigated the intervening factors during the middle postoperative period.

Therefore, the present study primarily aimed to evaluate the frequency of sleep disturbance at midterm after TKA in a Japanese cohort and to identify intervening factors for sleep, including knee pain and functional capability. We hypothesized that elevated sleep quality can be expected following a reduction in knee pain at midterm after TKA and that both knee pain and functional capability affect sleep quality even after TKA.

## Materials and methods

### Patients

This retrospective cohort study was conducted in accordance with the Strengthening the Reporting of Observational Studies in Epidemiology (STROBE) statement^24^ and was approved by institutional review board (IRB) of Kyushu University (approval no.: 2020-425). Written informed consent was obtained from the patients for their participation in this IRB-approved study. The data were handled while adhering to the ethical standards laid down in the Declaration of Helsinki.

A total of 500 patients who underwent primary TKA for knee OA at a single university hospital between April 2013 and August 2019 were recruited for this study. The inclusion criteria was as follows: (1) primary TKA performed with a parapatellar approach, (2) more than 18 months follow-up before the study. The sex ratio of the 500 patients recruited for this study was 15% and 85% for male and female, respectively. Out of these patients, 228 who underwent 281 TKAs responded to self-administered questionnaires. Worse Knee Society Score (KSS) 2011 was selected in patients who underwent bilateral TKA. Finally, 209 Japanese participants (35 males (17%), 168 females (80%), and 6 anonymous patients; mean age at the time of follow-up: 77.1 ± 8.3 years; mean body mass index [BMI]: 26 ± 4.2 kg/cm^2^) who provided with sufficient responses were included in this study. The mean follow-up period was 4.5 ± 1.9 years. Out of these 209 patients, 69 (33%) regularly took sleeping pills prescribed for sleep disturbance by a family doctor after the TKA.

### Surgical techniques

All TKAs were performed using the parapatellar approach, with a uniform protocol for postoperative rehabilitation^25–27^. The distal femoral cutting block was aligned using an intramedullary guide, whereas the proximal tibial cutting block was aligned using an extramedullary guide. The tibial resection surface was perpendicular to the predicted postoperative tibial mechanical axis, which was a line drawn from the center of the talar dome to the center of the resection surface. The rotational alignment was adjusted to the surgical epicondylar axis for the femoral component and to the medial third of the tibial tuberosity at the level of the patellar tendon attachment for the tibial component.

### Questionnaires

The Pittsburgh Sleep Quality Index (PSQI) is a multidisciplinary and internationally used 7-item questionnaire for the evaluation of sleep quality and disturbance^28,29^. Each item is scored on a scale from 0–3 summed up, and the maximum possible PSQI score is 21 points, with higher scores indicating worse outcomes and with sleep disturbance being defined using a cut-off value of > 5.5 points. High reproducibility and validity have been reported for the Japanese version of the PSQI (PSQI-J)^30^. A cut-off value of 5.5 points for sleep disturbance in the PSQI-J was used based on a previous validation study^30^.

Disease-specific patient-reported outcomes after TKA were assessed using the KSS 2011^31–33^. The subjective component of the KSS 2011 evaluates the patients’ symptoms, satisfaction, expectations, and functional activities, with a maximum possible score of 25 for “symptoms” (including 10 for pain during level walking), 40 for “patient satisfaction” (including 8 for pain level while lying in bed), 15 for “patient expectations”, 100 for “functional activities”, and 30 for “walking and standing”.

The Forgotten Joint Score (FJS)-12 is a 12-item questionnaire for the assessment of joint awareness in daily life^34–36^. The answers to 12 items concerning the frequency of joint awareness (never, 0 point; almost never, 1 point; seldom, 2 points; sometimes, 3 points; mostly, 4 points) are summed and converted into a 100-point scale, with higher scores indicating better outcomes. Question #1 pertains to awareness of the artificial joint in bed at night.

The 12-Item Short-Form Health Survey (SF-12) is a generic and well-established health-related QOL measure and consists of a subset of 12 items from the SF-36 scale^37,38^. Information from all 12 items is used to construct the Physical Component Summary, Mental Component Summary (MCS), and Role/Social Component Summary measures. In this study, mental QOL was evaluated using the SF-12 MCS.

### Statistical analyses

Continuous data are expressed as means ± standard deviations. Statistical analyses were performed using R software (The R Foundation for Statistical Computing, Vienna, Austria) and JMP Pro 15.1.0 (SAS Institute, Cary, North Carolina), with statistical significance set at a p-value of < 0.05. As the data were not missing completely at random, multiple imputation on the mice 3.13.0 package was applied with predictive mean matching^35,39^. A total of 100 imputed datasets were generated, and all missing values of other variables were imputed.

Welch’s t-test and the chi-squared test were used to compare patients with sleep disturbance (PSQI ≥ 5.5) and those without sleep disturbance (PSQI < 5.5)^30^. Power analyses indicated that, assuming a p-value of < 0.05 and a standard deviation of 23, a sample size of 169 knees would provide a statistical power of 80% for the detection of 10 points of functional activity category in the KSS 2011 among patients with and without sleep disturbance^32^. Spearman’s correlation was applied to examine the relationship between sleep quality (PSQI) and patient-reported outcomes (gait pain and functional activity category in the KSS 2011 as well as joint awareness in bed at night in the FJS-12). In order to determine the factors associated with the PSQI, multiple regression analyses with the stepwise variable entry method (adjusted R squared: 0.39) were performed using the following factors: patients’ demographics (age, sex, BMI, diagnosis, follow-up period, presence or absence of sleeping pill intake), satisfaction with pain level while lying in bed, pain during level walking, and functional activity category in the KSS 2011, awareness of the artificial joint in bed at night in the FJS-12, and SF-12 MCS.

### Ethical committee approval

Each author certifies that their institution approved the human protocol for this investigation and that the investigation was conducted in accordance with ethical principles in research.

## Results

The scores for the PSQI, pain during level walking, satisfaction with pain level while lying in bed, physical activity category in the KSS 2011, awareness of the artificial joint in bed at night in the FJS-12, and SF-12 MCS were 6.7 ± 3.0, 1.6 ± 2.3, 5.8 ± 1.8, 62 ± 22, 1.5 ± 1.4, and 56 ± 9.3 on average, respectively (see Appendix Supplementary data S1). Sleep disturbance (PSQI ≥ 5.5) occurred in 54% (113/209) of the participants.

**Figure 1:**
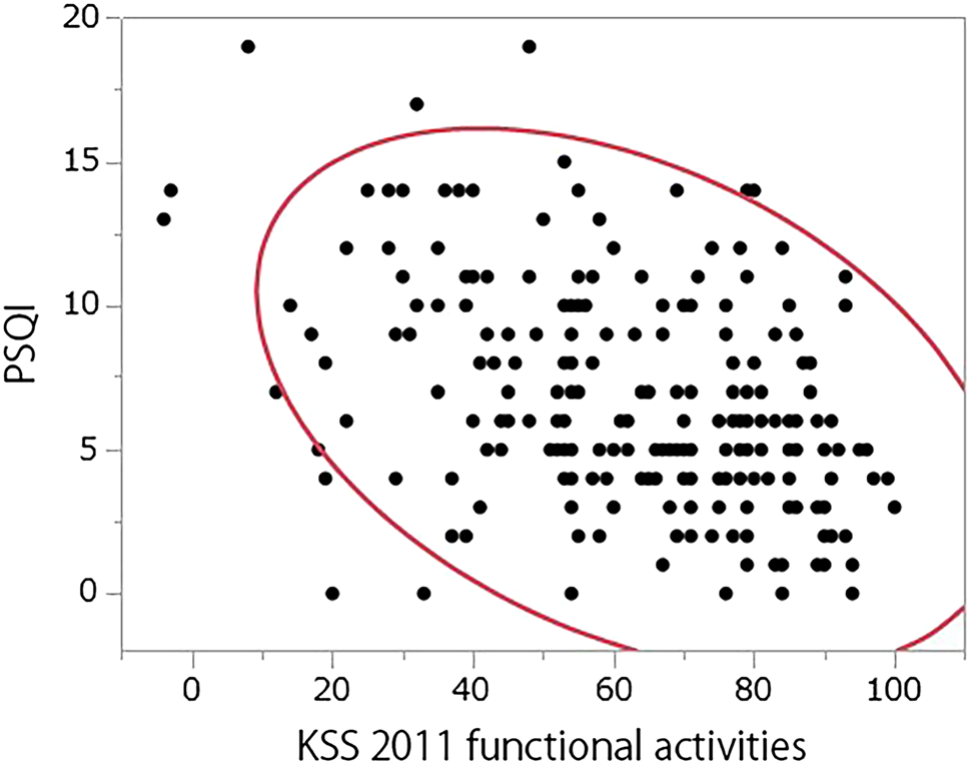
Significant correlation between the PSQI and functional activities in the KSS 2011 (p < 0.05). *PSQI* Pittsburgh sleep quality index, *KSS* knee society score.

Scores for pain during level walking, functional activities and joint awareness in bed at night were significantly higher in participants reporting good sleep quality (Table 1). Patient groups with and without sleep disturbance showed no significant differences with respect to age, BMI, follow-up duration, satisfaction with pain level while lying in bed, and SF-12 MCS (p > 0.05). Joint awareness during sleep (p < 0.01, ρ = − 0.28) and functional capability (p < 0.01, ρ = − 0.40) were significantly associated with better PSQI scores (Fig. 1). Pain when walking in the KSS 2011 showed no significant relationship with the PSQI (p > 0.05, ρ = 0.28). Multivariable analysis revealed that no sleeping pill intake, high functional capability, better mental QOL, and older age had a significant effect on sleep quality improvement (p < 0.05, Table 2). The normality of the residuals of the multiple regression model was confirmed (see Appendix Supplementary data S2).

**Table 1:** Comparison of patient demographics and patient-reported outcomes between groups with and without sleep disturbance.

	PSQI ≥ 5.5 (N = 113)	PSQI < 5.5 (N = 96)	p-value
Male/female, N (%)	14 (12%)/99 (88%)	21 (22%)/75 (78%)	0.02
Sleeping pill intake, N (%)	58 (51%)	10 (10%)	< 0.01
Pain during level walking in the KSS 2011 (0–10)	2.1 ± 1.0	0.9 ± 1.7	< 0.01
Functional activity category in the KSS 2011 (0–100)	56 ± 22	69 ± 19	< 0.01
Joint awareness during sleep in the FJS-12 (0–5)	1.9 ± 1.4	1.1 ± 1.2	< 0.01

Continuous data are expressed as mean ± standard deviation.

Age, body mass index, follow-up duration, sleep satisfaction in the KSS 2011, and SF-12 MCS showed no significant differences.

*PSQI* Pittsburgh sleep quality index, *KSS 2011* knee society score 2011, *FJS-12* forgotten joint score-12, *SF-12 MCS* 12-item short-form health survey mental component summary.

**Table 2:** Analysis of factors influencing the PSQI.

Factor	p-value	F-value	Positive effect
Sleeping pills	< 0.0001*	12.5	No intake
Functional activity category in the KSS 2011	< 0.0001*	5.5	High score
SF-12 MCS	0.01*	1.9	High score
Age	0.02*	1.7	Older age
Joint awareness during sleep in the FJS-12	0.07	1.2	
BMI	0.08	1.1	

*PSQI* Pittsburgh sleep quality index, *KSS 2011* knee society score 2011, *SF-12 MCS* 12-item short-form health survey mental component summary, *FJS-12* forgotten joint score-12, *BMI* body mass index.

*Statistically significant (p < 0.05).

## Discussion

The present study yielded the following most important findings: (1) the incidence of sleep disturbance was 54% at midterm after TKA in the Japanese cohort; (2) functional capability was a significant intervening factor associated with sleep quality; and (3) pain during level walking and joint awareness in bed at night were not identified as the reasons for sleep disturbance. These results suggest that functional capability bears an important relationship with sleep disturbance at midterm after TKA.

A Japanese epidemiological study conducted by Kim et al. reported an incidence of 30% for sleep disturbance^4^, and a meta-analysis showed an incidence of 17% for insomnia in Western patients who underwent total joint replacement^18,20^. After TKA, the incidence of insomnia was 1.8 times higher in Japanese patients even at midterm, as compared with that in the Japanese population-based cohort^4^. Furthermore, our study results indicated that sleep disturbance occurred in 54% of Japanese patients after TKA, which is a higher rate than those reported by previous studies in Western patients^16–20,23^. This is the first study to evaluate the frequency of sleep disturbance in Asian cohorts after TKA. The sleep quality was lower (6.7 vs. 2.1^23^), the incidence of sleep disturbance was higher (54% vs. 14%^23^), and the rate of sleeping pill intake was higher (33% vs. 18%^19^) in this study than in previous reports^19,23^. The reason for these differences in this and previous studies could be not residual pain after TKA, but both reduced functional capability and physical activities.

In the present study, we particularly focused on intervening factors, especially residual knee pain and reduced functional capability after TKA. Then, functional capability was positively correlated with better sleep quality and was identified as one of the positive factors for sleep quality improvement. It is well recognized that physical activity results in better sleep quality, and a quick and efficient replenishment for the body request^40,41^. In Japan, 40% of elderly continue to perform physical activities for 30 min more than twice a week, which is not enough and action assignment [42,44]. Compared to healthy elderly patients, patients with OA exhibit decreased physical activity levels^43^. As reported by a previous study, 62% of patients with lower-extremity OA do not achieve the recommended physical activity levels^44^. Even in patients with OA, moderate-intensity physical activity corresponding to 3–5.9 metabolic equivalents (METs) contributes to the extension of healthy life expectancy^45^. Previous studies reported that the objective activity level did not improve much after surgery in TKA patients and that physical activity decreased with age^46^. In this study, patients who reported functional capability after TKA showed lower scores than healthy Asian cohorts in their 80 s (62 vs. 74)^47^. Considering the harmful effects of sleep disorder in the elderly, a non-drug approach (e.g., proactive intervention) is required to increase their habitual physical activity levels and is a priority for elderly patients with insomnia. In this study, one third of patients regularly took sleeping pills even after TKA. Recently, digital patient engagement has been suggested as a strategy for increasing the physical activity levels among patients^48^. Bäcker et al. described that an app-based knee trainer was a promising tool for improving the functional outcomes after TKA, including the KSS function score^49^.

There are conflicting reports on the relationship between sleep quality and pain scores in arthroplasty patients^15,16^. Previous reports suggested that pain was the main cause of sleep disturbance^50^ and that poor sleep quality was reciprocally associated with increased pain perception^51^ and insufficient exercise^4^. This study showed that knee pain was restored at midterm after TKA; however, their activities of daily living and sleep quality were impaired.

In this study, better mental QOL and older age were associated factors for better sleep quality. A previous study reported that anxious, depressed, or pain-catastrophizing patients who underwent TKA had inferior preoperative and postoperative pain and function^52^. Long et al. also reported that mental elements were factors affecting sleep quality^19^. Yoshiuchi et al. suggested 4000–5000 steps/day and/or 5–7.5 min/day for impaired mental and psychosocial health, including a depressed mood state^53^. Canfield et al. provided evidence that nonpharmacologic interventions, such as self-guided meditation, might aid in improving the sleep quality during the perioperative period^22^. Objectively measured age-related changes in sleep in a Japanese cohort indicated a longer sleep duration and equivalent sleep efficacy in those aged > 60 years^54^, which is consistent with our findings.

This study has some limitations. First, this study was a retrospective analysis, making it susceptible to potential bias. Second, unreturned questionnaire data of 54% of the Japanese participants impacted the sample size of this study. The low response rate and sex imbalance in the sample population would bias the results. Although this low rate was similar to that of previous studies^32,35^, a higher response rate with longer follow-up is needed. The study was limited by the small number of male subjects as the male-to female ratio was approximately 1:6. However, the Japanese Orthopaedic Association National Registry (JOANR) reported that 18% and 82% of males and females, respectively, undertook TKA in 2021^55^, which is consistent with this study. Third, the physical activity or activity level were not objectively measured in this study. Although the measurement tools used in this study: patients-reported outcome measures (PROMs) cannot provide physical activity during daily living, higher functional capacity may^56^ or may not be^57^ related to increased engagement in physical activity. Further studies evaluating the effect of interventions are necessary to encourage daytime exercises. Finally, the minimum clinically important difference for each outcome (SF-12, Join awareness during sleep, physical activity) was not a priori stablished and interpreted for between groups comparisons (with vs without sleep disturbance). However, the results of multiple regression analyses are central to the conclusion of the present study.

## Conclusions

This study investigated the sleep quality, incidence of sleep disturbance, and influencing factors at midterm after TKA in Japanese patients. In this study, the incidence of sleep disturbance was 54%, and functional capability was a significant positive factor interacting with sleep quality. Knee pain did not affect the sleep quality at midterm after TKA; in contrast, no sleeping pill intake, better mental QOL, and older age were positive factors for achieving improved sleep quality.

### Supplementary Information

Supplementary Information.

## Data Availability

The datasets used and/or analysed during the current study available from the corresponding author on reasonable request.

## References

[1] Tan JS, Tikoft E, O'Sullivan P, Smith A, Campbell A, Caneiro JP, Kent P (2021). The relationship between changes in movement and activity limitation or pain in people with knee osteoarthritis: A systematic review. J. Orthop. Sports Phys. Ther..

[2] Zhang Y, Jordan JM (2010). Epidemiology of osteoarthritis. Clin. Geriatr. Med..

[3] Muraki S, Akune T, Oka H, Ishimoto Y, Nagata K, Yoshida M, Tokimura F, Nakamura K, Kawaguchi H, Yoshimura N (2012). Incidence and risk factors for radiographic knee osteoarthritis and knee pain in Japanese men and women: A longitudinal population-based cohort study. Arthritis Rheum..

[4] Kim K, Uchiyama M, Okawa M, Liu X, Ogihara R (2000). An epidemiological study of insomnia among the Japanese general population. Sleep..

[5] Dai Z, Neogi T, Brown C, Nevitt M, Lewis CE, Torner J, Felson DT (2020). Sleep quality is related to worsening knee pain in those with widespread pain: The multicenter osteoarthritis study. J. Rheumatol..

[6] Itani O, Kaneita Y, Munezawa T, Mishima K, Jike M, Nakagome S, Tokiya M, Ohida T (2016). Nationwide epidemiological study of insomnia in Japan. Sleep Med..

[7] Ohtsuki M, Wakasugi Y, Narukawa T, Uehara S, Ohkubo T (2021). Are lifestyle factors significantly associated with self-rated health among Japanese female healthcare students?. BMC Public Health..

[8] Smith MT, Finan PH, Buenaver LF, Robinson M, Haque U, Quain A, McInrue E, Han D, Leoutsakis J, Haythornthwaite JA (2015). Cognitive-behavioral therapy for insomnia in knee osteoarthritis: A randomized, double-blind, active placebo-controlled clinical trial. Arthritis Rheumatol..

[9] Cruz-Almeida Y, King CD, Goodin BR, Sibille KT, Glover TL, Riley JL (2013). Psychological profiles and pain characteristics of older adults with knee osteoarthritis. Arthritis Care Res. (Hoboken).

[10] Ritter MA, Keating EM, Sueyoshi T, Davis KE, Barrington JW, Emerson RH (2016). Twenty-five-years and greater, results after nonmodular cemented total knee arthroplasty. J. Arthroplast..

[11] Matsushita Y, Hamai S, Okazaki K, Murakami K, Ma Y, Kiyohara M, Mizu-Uchi H, Akasaki Y, Nakashima Y (2018). Recreational sports, workout and gym activities after total knee arthroplasty: Asian cohort study. J. Orthop..

[12] Crawford DA, Adams JB, Hobbs GR, Berend KR, Lombardi AV (2020). Higher activity level following total knee arthroplasty is not deleterious to mid-term implant survivorship. J. Arthroplast..

[13] Kiyohara M, Hamai S, Okazaki K, Fujiyoshi D, Mizu-Uchi H, Nakashima Y (2022). Evaluation of the balance function before and after total knee arthroplasty using Berg balance scale. Arch. Orthop. Trauma Surg..

[14] Inui H, Yamagami R, Kono K, Kawaguchi K (2023). What are the causes of failure after total knee arthroplasty?. J. Joint Surg. Res..

[15] Er MS, Altinel EC, Altinel L, Erten RA, Eroglu M (2014). An assessment of sleep quality in patients undergoing total knee arthroplasty before and after surgery. Acta Orthop. Traumatol. Turc..

[16] Chen AF, Orozco FR, Austin LS, Post ZD, Deirmengian CA, Ong AC (2016). Prospective evaluation of sleep disturbances after total knee arthroplasty. J. Arthroplast..

[17] Manning BT, Kearns SM, Bohl DD, Edmiston T, Sporer SM, Levine BR (2017). Prospective assessment of sleep quality before and after primary total joint replacement. Orthopedics.

[18] Scott JE, Mathias JL, Kneebone AC (2015). Incidence of delirium following total joint replacement in older adults: A meta-analysis. Gen. Hosp. Psychiatry..

[19] Long G, Suqin S, Hu Z, Yan Z, Huixin Y, Tianwang L, Yang Y, Zhenhu W (2019). Analysis of patients' sleep disorder after total knee arthroplasty—A retrospective study. J. Orthop. Sci..

[20] Rong X, Ding ZC, Yu HD, Yao SY, Zhou ZK (2021). Risk factors of postoperative delirium in the knee and hip replacement patients: A systematic review and meta-analysis. J. Orthop. Surg. Res..

[21] Van Meirhaeghe JP, Salmon LJ, O'Sullivan MD, Gooden BR, Lyons MC, Pinczewski LA, Roe JP (2021). Improvement in sleep patterns after hip and knee arthroplasty: A prospective study in 780 patients. J. Arthroplast..

[22] Canfield MJ, Cremins MS, Vellanky SS, Teng R, Belniak RM (2021). Evaluating the success of perioperative self-guided meditation in reducing sleep disturbance after total knee arthroplasty. J. Arthroplast..

[23] Mukartihal RK, Angadi DS, Mangukiya HJ, Singh NK, Varad S, Ramesh PA, Patil SS (2022). Temporal changes in sleep quality and knee function following primary total knee arthroplasty: A prospective study. Int. Orthop..

[24] von Elm E, Altman DG, Egger M, Pocock SJ, Gøtzsche PC, Vandenbroucke JP (2007). The strengthening the reporting of observational studies in epidemiology (STROBE) statement: Guidelines for reporting observational studies. Lancet..

[25] Hamai S, Miura H, Higaki H, Matsuda S, Shimoto T, Sasaki K, Yoshizumi M, Okazaki K, Tsukamoto N, Iwamoto Y (2008). Kinematic analysis of kneeling in cruciate-retaining and posterior-stabilized total knee arthroplasties. J. Orthop. Res..

[26] Hamai S, Miura H, Okazaki K, Shimoto T, Higaki H, Iwamoto Y (2014). No influence of coronal laxity and alignment on lift-off after well-balanced and aligned total knee arthroplasty. Knee Surg. Sports Traumatol. Arthrosc..

[27] Hamai S, Okazaki K, Shimoto T, Nakahara H, Higaki H, Iwamoto Y (2015). Continuous sagittal radiologic evaluation of stair-climbing in cruciate-retaining and posterior-stabilized total knee arthroplasties. J. Arthroplast..

[28] Buysse DJ, Reynolds CF, Monk TH, Berman SR, Kupfer DJ (1989). The Pittsburgh Sleep Quality Index: A new instrument for psychiatric practice and research. Psychiatry Res..

[29] Pilz LK, Keller LK, Lenssen D, Roenneberg T (2018). Time to rethink sleep quality: PSQI scores reflect sleep quality on workdays. Sleep..

[30] Doi Y, Minowa M, Uchiyama M, Okawa M, Kim K, Shibui K, Kamei Y (2000). Psychometric assessment of subjective sleep quality using the Japanese version of the Pittsburgh Sleep Quality Index (PSQI-J) in psychiatric disordered and control subjects. Psychiatry Res..

[31] Noble PC, Scuderi GR, Brekke AC, Sikorskii A, Benjamin JB, Lonner JH, Chadha P, Daylamani DA, Scott WN, Bourne RB (2012). Development of a new knee society scoring system. Clin. Orthop. Relat. Res..

[32] Matsuda S, Kawahara S, Okazaki K, Tashiro Y, Iwamoto Y (2013). Postoperative alignment and ROM affect patient satisfaction after TKA. Clin. Orthop. Relat. Res..

[33] Nishitani K, Nakamura S, Kuriyama S (2023). Clinical evaluation of knee joint diseases. J. Joint Surg. Res..

[34] Behrend H, Giesinger K, Giesinger JM, Kuster MS (2012). The "forgotten joint" as the ultimate goal in joint arthroplasty: Validation of a new patient-reported outcome measure. J. Arthroplast..

[35] Yamate S, Hamai S, Kawahara S, Hara D, Motomura G, Ikemura S, Fujii M, Sato T, Harada S, Harada T, Kokubu Y, Nakashima Y (2022). Multiple imputation to salvage partial respondents: Analysis of the forgotten joint score-12 after total hip arthroplasty. J. Bone Joint Surg. Am..

[36] Yamate S, Hamai S, Lyman S, Konishi T, Kawahara S, Yamaguchi R, Hara D, Motomura G (2023). Clinical evaluation of hip joint diseases: total hip arthroplasty to support patients’ quality of life. J. Joint Surg. Res..

[37] Tan, M. L. S., Wee, H. L., Salim, A., Lee, J., Ma, S., & Heng, D. Validity of a revised short form-12 health survey version 2 in different ethnic populations. Validity a Revis Short Form-12 Heal Surv Version 2 Differ Ethn Popul. 169608. 27412055

[38] Ware J, Kosinski M, Keller SD (1996). A 12-Item Short-Form Health Survey: Construction of scales and preliminary tests of reliability and validity. Med. Care..

[39] Rubin DB (1976). Inference and missing data. Biometrika..

[40] Park I, Díaz J, Matsumoto S, Iwayama K, Nabekura Y, Ogata H, Kayaba M, Aoyagi A, Yajima K, Satoh M, Tokuyama K, Vogt KE (2021). Exercise improves the quality of slow-wave sleep by increasing slow-wave stability. Sci. Rep..

[41] Sullivan Bisson AN, Robinson SA, Lachman ME (2019). Walk to a better night of sleep: Testing the relationship between physical activity and sleep. Sleep Health..

[42] Makizako H, Nakai Y, Shiratsuchi D, Akanuma T, Yokoyama K, Matsuzaki-Kihara Y, Yoshida H (2021). Perceived declining physical and cognitive fitness during the COVID-19 state of emergency among community-dwelling Japanese old-old adults. Geriatr. Gerontol. Int..

[43] Rosemann T, Gensichen J, Sauer N, Laux A, Szecsenyi J (2007). The impact of concomitant depression on quality of life and health service utilisation in patients with osteoarthritis. Rheumatol. Int..

[44] Fontaine KR, Heo M, Bathon J (2004). Are US adults with arthritis meeting public health recommendations for physical activity?. Arthritis Rheum..

[45] McAlindon TE, Wilson PW, Aliabadi P, Weissman B, Felson DT (1999). Level of physical activity and the risk of radiographic and symptomatic knee osteoarthritis in the elderly: The Framingham study. Am. J. Med..

[46] Frimpong E, van der Jagt DR, Mokete L, Pietrzak J, Kaoje YS, Smith A, McVeigh JA, Meiring RM (2020). Improvements in objectively measured activity behaviors do not correlate with improvements in patient-reported outcome measures following total knee arthroplasty. J. Arthroplast..

[47] Taniguchi N, Matsuda S, Kawaguchi T, Tabara Y, Ikezoe T, Tsuboyama T, Ichihashi N, Nakayama T, Matsuda F, Ito H (2015). The KSS 2011 reflects symptoms, physical activities, and radiographic grades in a Japanese population. Clin. Orthop. Relat. Res..

[48] Knapp PW, Keller RA, Mabee KA, Pillai R, Frisch NB (2021). Quantifying patient engagement in total joint arthroplasty using digital application-based technology. J. Arthroplast..

[49] Bäcker HC, Wu CH, Schulz MRG, Weber-Spickschen TS, Perka C, Hardt S (2021). App-based rehabilitation program after total knee arthroplasty: A randomized controlled trial. Arch. Orthop. Trauma Surg..

[50] Amaro-Díaz L, Montoro CI, Fischer-Jbali LR, Galvez-Sánchez CM (2022). Chronic pain and emotional stroop: A systematic review. J. Clin. Med..

[51] Alexandre C, Latremoliere A, Ferreira A, Miracca G, Yamamoto M, Scammell TE, Woolf CJ (2017). Decreased alertness due to sleep loss increases pain sensitivity in mice. Nat. Med..

[52] Wood TJ, Gazendam AM, Kabali CB (2021). Postoperative outcomes following total hip and knee arthroplasty in patients with pain catastrophizing, anxiety, or depression. J. Arthroplast..

[53] Yoshiuchi K, Nakahara R, Kumano H, Kuboki T, Togo F, Watanabe E, Yasunaga A, Park H, Shephard RJ, Aoyagi Y (2006). Yearlong physical activity and depressive symptoms in older Japanese adults: Cross-sectional data from the Nakanojo study. Am. J. Geriatr. Psychiatry..

[54] Aoyagi Y, Park S, Cho S, Shephard RJ (2018). Objectively measured habitual physical activity and sleep-related phenomena in 1645 people aged 1–91 years: The Nakanojo Community Study. Prev. Med. Rep..

[55] The Japanese Society for Replacement Arthroplasty [JSRA]: https://jsra.info/en/.

[56] Fujita T, Hamai S, Shiomoto K, Okazawa K, Nasu Y, Hara D, Harada S, Motomura G, Ikemura S, Fujii M, Kawahara S, Kawaguchi K, Nakashima Y (2022). Analysis of factors influencing patient satisfaction after total hip arthroplasty in a Japanese cohort: A significant effect of postoperative physical activity. J. Phys. Ther. Sci..

[57] Sandell Jacobsen J, Thorborg K, Hölmich P, Bolvig L, Storgaard Jakobsen S, Søballe K, Mechlenburg I (2018). Does the physical activity profile change in patients with hip dysplasia from before to 1 year after periacetabular osteotomy?. Acta Orthop..

