# Supplementary material for: Interaction between functional capability and sleep quality at midterm after total knee arthroplasty: a Japanese retrospective cohort study
Source: Sci Rep. 2023 Oct 26;13:18373. doi: 10.1038/s41598-023-45603-4 (PMC10603171; doi:10.1038/s41598-023-45603-4)
Supplement: Supplementary file 1 — Supplementary Information. [file 41598_2023_45603_MOESM1_ESM.docx]

**Supplementary data 1.** The normality of the residuals of the multiple regression model was confirmed.


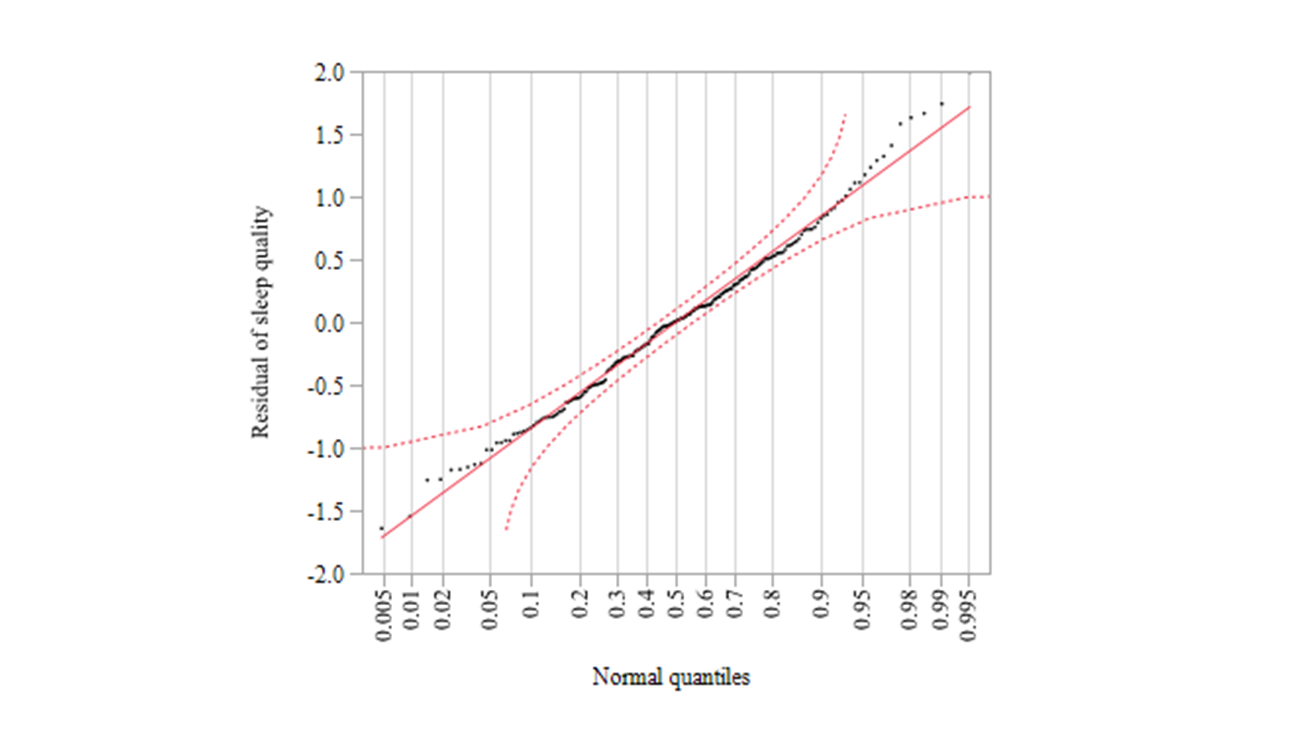


**Supplementary data 2.** Demographic data and patient-reported outcomes for the study cohort.

| 209 patients | |
| --- | --- |
| Age at surgery#, y | 77.1 ± 8.3 |
| Sex (male; female: anonymous), knees | 35;168:6 |
| Body mass index#, kg/m^2^ | 26 ± 4.2 |
| Follow-up#, y | 4.5 ± 1.9 |
| Pain during level walking in the KSS 2011# | 1.5 ± 2.3 |
| Functional activity category in the KSS 2011＃ | 63 ± 21.5 |
| FJS-12# | 48.3 ± 27 |
| The 12-Item Shor-Form Health Survey Physical Component Summary# | 35.2 ± 12.5 |
| The 12-Item Short-Form Health Survey Role/Social Component Summary# | 41.6 ± 15.6 |
| The 12-Item Short-Form Health Survey Mental Component Summary# | 56 ± 9.3 |

#The values are given as the mean ± standard deviations.

Abbreviations: KSS 2011, Knee Society Score 2011; FJS-12, Forgotten Joint Score-12; SF-12, The 12-Item Short-Form Health Survey; PCS, Physical Component Summary; RCS, Role/Social Component Summary.
